# Development of a Simultaneous Quantification Method for Multiple Modes of Nitrogen in Leaf Models Using Near-Infrared Spectroscopic Measurement

**DOI:** 10.3390/s24041160

**Published:** 2024-02-09

**Authors:** Atsushi Hashimoto, Ken-ichiro Suehara, Takaharu Kameoka

**Affiliations:** 1 Graduate School of Bioresources, Mie University, 1577 Kurimamachiya-cho, Tsu 514-8507, Japan; kameoka@mie-u.ac.jp; 2 Graduate School of Regional Innovation Studies, Mie University, 1577 Kurimamachiya-cho, Tsu 514-8507, Japan; suehara@bio.mie-u.ac.jp; 3 Research Center for Social Systems, Shinshu University, 5304-6 Nagakura, Karuizawa 389-0111, Japan

**Keywords:** infrared spectroscopy, nitrate nitrogen, proteinic nitrogen, leaf model, nitrogen, FT-NIR, mid-infrared spectroscopy

## Abstract

By focusing our attention on nitrogen components in plants, which are important for cultivation management in data-driven agriculture, we developed a simple, rapid, non-chemical and simultaneous quantification method for proteinic and nitrate nitrogen in a leaf model based on near-infrared (NIR) spectroscopic information obtained using a compact Fourier Transform NIR (FT-NIR) spectrometer. The NIR spectra of wet leaf models impregnated with a protein–nitric acid mixed solution and a dry leaf model obtained by drying filter paper were acquired. For spectral acquisition, a compact MEMS (Micro Electro Mechanical Systems) FT-NIR spectrometer equipped with a diffuse reflectance probe accessory was used. Partial least square regression analysis was performed using the spectral information of the extracted absorption bands based on the determination coefficients between the spectral absorption intensities and the contents of the two-dimensional spectral analysis between NIR and mid-infrared spectral information. Proteinic nitrogen content in the dry leaf model was well predicted using the MEMS FT-NIR spectroscopic method. Additionally, nitrate nitrogen in the dry leaf model was also determined by the provided method, but the necessity of adding the data for a wider range of nitric acid concentrations was experimentally indicated for the prediction of nitrate nitrogen content in the wet leaf model. Consequently, these results experimentally suggest the possibility of the application of the compact MEMS FT-NIR for obtaining the bioinformation of crops at agricultural on-sites.

## 1. Introduction

For data-driven agriculture, which aims at the sustainable production of high-quality agricultural products, the technological development of a simple, easy, rapid, and non-chemical measurement method for biological information and agricultural on-site use for the acquisition of the bioinformation of target crops is desirable. Such types of research aiming to obtain the bioinformation of target crops in agricultural fields for cultivation management have been conducted [1,2,3,4,5]. However, such technical development has not been significant in comparison to that in the measurement of environmental information using sensor networks [6,7,8,9,10]. For these measurements, easy, rapid, non-chemical, and efficacious ICT system technique, are usually needed; thus, the application of optical (spectroscopic) sensing techniques is desirable.

Research on obtaining plant vigor information has been actively conducted using electromagnetic waves in various wavelength ranges; this has included using machine vision for color image analysis for the purpose of cultivation and food process management [11,12,13,14,15,16]. For example, Lee et al. developed a method for measuring spatial variability in the field for crop production by combining machine vision and thermographic techniques [17]. Moreover, color machine vision techniques have been actively applied to measure plant vigor in the field for agricultural and food processes [18,19,20,21]. Additionally, Kameoka et al. described a method to apply optically sensed bioinformation to the cultivation process [22]. Furthermore, it was suggested that these sensing methods could have a close affinity with information technologies [23]. We have also been conducting research on multiband optical sensing to comprehensively acquire bioinformation relating to the elemental balance, organic components, and color appearance changes formed by the overall metabolic process [13,24]; we experimentally demonstrated the mid-infrared (MIR) spectroscopic determination of the nitrate nitrogen in fresh leaves [25]. In addition, it was found that the developed XRF spectroscopic method was useful as a quantitative analytical method for the elemental contents of the fresh leaf by comparing it with a standardly used Inductively Coupled Plasma method [26]. These results could suggest the importance of accumulating quantitative spectral information relating to the organic components, such as the saccharides and organic acids, of the fresh leaf [12,24].

On the other hand, advances in semiconductor technology have led to the development of compact and simple optical measurement instruments, which are expected to have the potential for measurement in the field. For example, compact MEMS FT-NIR (near-infrared) spectrometers that apply semiconductor microfabrication technology have become commercially available, and the application of the FT-NIR system for obtaining bioinformation for the purpose of cultivation management has been described [27,28]. However, it can be difficult to quantitatively analyze NIR spectral features because of the weak absorption and complexity of the absorption bands in comparison to MIR spectral features.

In addition, nitrogen components have a significant impact on agriculture and cultivation. In this sense, the nitrogen in agricultural crops is a simple parameter. Hence, rapid measurement methods are becoming important. Moreover, the multiple modes of nitrogen in plants are especially important for cultivation management. As already described, the simple and rapid measurement of nitrogen characteristics in plants in the field is desired for both environmental and agricultural purposes. This study aimed to develop a simultaneous quantification method for nitrate and protein nitrogen in a leaf model based on NIR spectroscopic information obtained using a compact FT-NIR since nitrate nitrogen content in leaves could represent information about plant vigor use. For developing the NIR spectroscopic determination method for the multiple modes of nitrogen contents in fresh leaves, it is important to acquire spectral information of fresh leaves with various concentrations of multiple modes of nitrogen and various combinations of the multiple modes of nitrogen. However, it could be impossible to collect such types of actual fresh leaves [29]. In this study, we tried to construct leaf models for NIR spectral analysis. To construct stable models for the determination of the multiple modes of nitrogen based on their absorption bands in the NIR region where the complicated overlapped absorption bands in the MIR region are observed, we performed a two-dimensional spectral analysis between the NIR spectroscopic information of the leaf models with the chemically and geometrically complicated structures and the MIR spectroscopic information of the nitrogen mixture solutions as the homogeneous sample because the NIR spectral features could represent the complex mixed absorption bands such as the overtones of the fundamental vibrational modes of molecules observed in the MIR region.

## 2. Materials and Methods

### 2.1. Materials

#### 2.1.1. Leaf Model Solution

Leaf Model Solution, the aqueous solution of the chemical reagent mixture composed of proteinic and nitrate nitrogen, was prepared for understanding the NIR spectral features of the multiple modes of nitrogen. Casein and nitrate acid were used as the proteinic and nitrate nitrogen, respectively. Casein, which contains about 15% nitrogen, was dissolved in a 0.1 N sodium hydroxide solution to obtain a solution containing about 0.6% proteinic nitrogen to prepare the standard leaf model solution. Additionally, by increasing or decreasing the casein and nitrate concentrations up to nine levels, nitrogen mixture solutions with different ratios of nitrate nitrogen to proteinic content were prepared. For the preparation of leaf model solutions, all reagents were obtained from the FUJIFILM Wako Pure Chemical Corporation, Osaka, Japan.

#### 2.1.2. Wet Leaf Model

**Figure 1:**
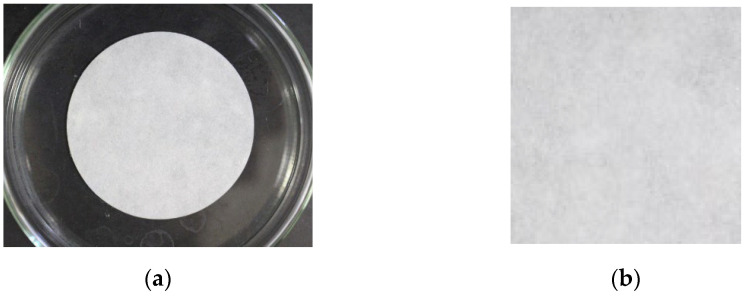
Photograph of a leaf model. (**a**) Overview; (**b**) magnified view.

Filter paper (55 mmϕ, No.1, Advantec Toyo Kaisha, Ltd., Tokyo, Japan) placed in a petri dish (90 mmϕ) was impregnated with 600 μL of the above leaf model solution of the specified concentrations of casein and nitrate acid, whose weight was measured. After the impregnation, the filter paper was kept in the petri dish with a lid on it for about 15 min at room temperature (298 K) to homogenize the leaf model distribution. The filter comprised polysaccharide and fresh leaf components with a complicated geometrical structure as shown in Figure 1. After measuring the weight to obtain the water content, the spectral information was acquired.

#### 2.1.3. Dry Leaf Model

The wet leaf model, after homogenizing the distribution of the leaf model solution, was dried in an oven at 353 K for 3 d until the weight change was negligible. After cooling the dry material, it was kept in a desiccator at room temperature (298 K), and the dry leaf model for the spectral measurements was prepared. The concentrations of the casein and nitric acid were then concentrated in the paper filter with the complicated geometrical structure.

### 2.2. Methods

#### 2.2.1. NIR Spectroscopic Measurements

**Figure 2:**
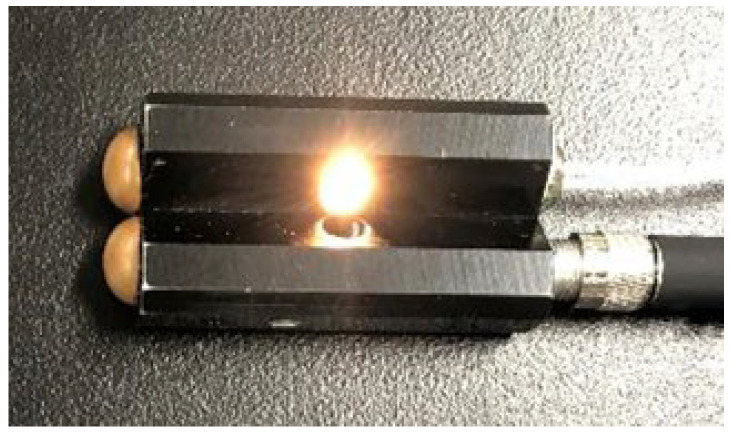
Photographs of diffuse reflection attachment for the FT-NIR system.

A compact MEMS FT-NIR (C15511-01, Hamamatsu Photonics K.K., Hamamatsu, Japan) equipped with a diffuse reflectance (DR) probe displayed in Figure 2 was used for the NIR spectral acquisition of the two types of leaf models. The DR probe was custom-made by Dunamist Co., Ltd. (Hamamatsu, Japan), and a 5 W halogen lamp was equipped with the attachment. The optical interferometer of the FT-NIR system includes an optical input section, beam splitter, fixed mirror, movable mirror (3 mmϕ), and a built-in light detector. The light detector acquires the intensity signal, which varies with the position of the movable mirror. By processing this intensity signal through computational operations (Fourier transform), spectral information is obtained. It was connected to a PC via a USB. The optical interferometer is equipped with a 3 mmϕ movable mirror using MEMS technology. The movable and fixed mirrors of the optical interferometer are compactly and robustly mounted. For the reference as the diffuse reflector, a white reflector (1001DP colorimeter, Nippon Denshoku Industries Co., Ltd., Tokyo, Japan) was used.

After the light source was stabilized, the white reflector was placed on the DR probe and the reference measurement was performed. The leaf model was then placed in the center of the probe, and the white reflector was placed on it to measure the sample spectrum. Before the diffusion spectral measurement of each leaf model, the reference measurement was performed. One hundred and twenty-eight scans of symmetrical interferograms at about a 6 cm^−1^ resolution were co-added for each spectrum. The NIR spectra of the leaf models were obtained from 9000 to 4000 cm^−1^. All the NIR spectra were measured at 298 K. The spectral acquisitions were repeated six times. In addition, the NIR spectra of the actual fresh leaves were also acquired by the same method as that for the leaf models.

#### 2.2.2. MIR Spectroscopic Measurements

A Fourier transform infrared spectrometer (FT-IR; Spectrum 400, PerkinElmer, Waltham, MA, USA) equipped with an ATR sampling accessory (Durasampl IR, Applied System, Smiths Detection Inc., Danbury, CT, USA) with a diamond internal reflection element (IRE) was used to collect the MIR spectra of the leaf model solution. A drop of the solutions was placed on the IRE crystal and the spectra were collected. The instrument was purged to minimize the spectral contribution due to atmospheric water vapor. Sixty-four scans of symmetrical interferograms at a 4 cm^−1^ resolution were co-added for each spectrum. The MIR spectra of the aqueous solutions were obtained from 4000 to 800 cm^−1^. All the MIR spectra were measured at 298 K.

#### 2.2.3. Analytical Methods

- Spectral treatments

Near-infrared spectra have a weak absorption, and it is difficult to capture slight differences due to large peaks such as with water. Therefore, in this study, when using the second derivative spectrum by the Savitzky–Golay method [30] to remove the noise, we first examined the number of smoothing points. The second-order polynomial and 11- or 17-point windows were used for the NIR spectra of the leaf models and the MIR spectra of the leaf model solutions, respectively.

- Two-dimensional spectral analysis

To evaluate the relationship between the NIR spectra of the leaf models and the MIR spectra of the leaf model solutions, two-dimensional correlation spectroscopic analysis [31,32] was performed using 2DShige software version 1.3 [33]. The information relating to the casein or nitrate acid concentration was analyzed.

- PLSR analysis

A partial least squares regression (PLSR) applied in Unscrambler version 10.2 (CAMO Software, Oslo, Norway) was utilized to establish calibrations between the diffuse reflectance spectra of the leaf models and the nitrogen concentrations. A leave One-Out (LOO) cross validation technique was performed. The use of Unscrambler software was able to determine the recommended number of factors to reduce the prediction error. The final calibration model was acquired from the full data employing these factors’ numbers.

## 3. Results and Discussion

### 3.1. NIR Spectral Characteristics of Leaf Models Using Filter Paper as Structural Part of the Leaf Model

#### 3.1.1. NIR Spectral Characteristics of Leaf Models

**Figure 3:**
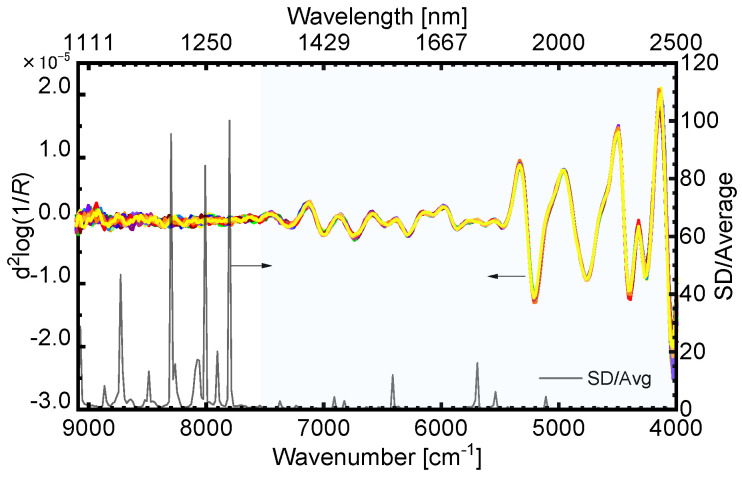
Influences of number of spectral measurements on second derivative spectral feature differences for filter paper as supporting material for leaf models and standard deviation spectra. The black line means the standard deviation divided by the average of the second derivative value, and the different colored lines indicate the twenty second derivative NIR spectra.

Figure 3 shows the second derivative NIR spectra when one dry paper filter without any impregnation was measured twenty times. Strong absorption bands were observed around 6700, 5200, 4700, 4400, and 4200 cm^−1^. On the other hand, in the region of 9000–7500 cm^−1^ where the second overtone of the fundamental vibrations of the functional group appears, variations between second derivative values were observed, and almost no significant absorption band was confirmed in this region. Additionally, in Figure 3, the spectrum of the standard deviation divided by the average of the second derivative value is displayed. The values were extremely low where the significant absorption bands were observed in the region of 9000–7500 cm^−1^, and significant values were confirmed in the region of 9000–7500 cm^−1^. Based on these results, we decided to proceed with the analysis focusing on the spectral information in the region of 7500–4000 cm^−1^.

**Figure 4:**
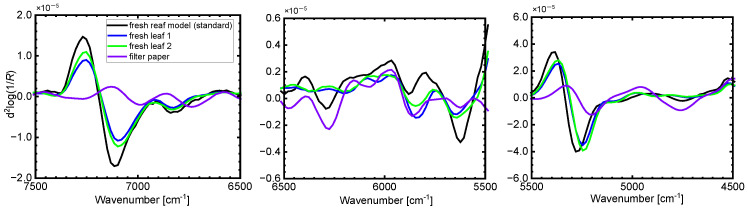
Comparison of the second derivative spectrum of the wet leaf model containing a standard concentration of proteinic and nitrate nitrogen to those of the paper filter and actual fresh leaves.

Figure 4 shows the second derivative spectra of the wet leaf model with the standard concentrations of proteinic and nitrate nitrogen by comparing to those of the actual fresh leaves and the wet leaf model containing standard concentration of proteinic and nitrate nitrogen. The spectrum of the wet leaf model prepared by impregnating the leaf model solution with the standard concentrations of proteinic and nitrate nitrogen into the filter paper indicated features similar to those of the actual fresh leaves. In addition, the strong absorption bands characterizing the wet leaf model and the actual leaves were observed around 7150, 6800, 6300, 5800, 5600, 5250 cm^−1^. These results experimentally suggested that the proposed leaf models could be acceptable for the samples for developing the NIR spectroscopic quantification method of the multiple modes of nitrogen in fresh leaves with chemically and geometrically complicated structures. Furthermore, using such leaf models, the chemical and water contents could be controlled. This point is especially important for developing the NIR spectroscopic quantification method.

#### 3.1.2. Nitrogen Content Determination Using the PRSR Method

**Figure 5:**
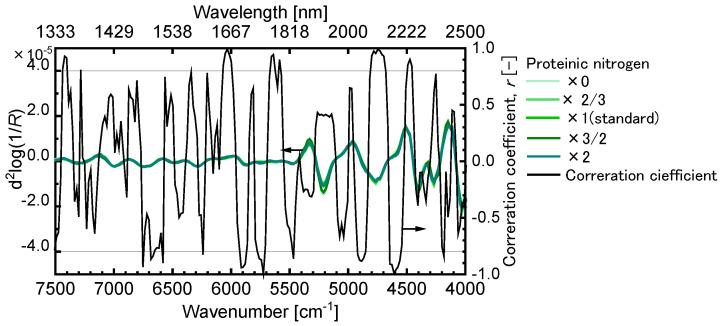
Second derivative spectra of dry leaf models with a standard concentration of nitrate acid and correlation coefficient spectrum between those and the proteinic nitrogen concentration.

Figure 5 represents the second derivative spectra of the dry leaf models with a standard concentration of nitrate acid and correlation coefficient spectrum between those and the proteinic nitrogen concentration. The spectral pattern variations between the samples were almost negligible in the vertical range shown in Figure 5, though the correlation coefficients between the casein contents and the second derivatives indicated high values for many wavenumbers. In addition, the strong peaks characterizing the dry leaf models were observed around 5200, 4800, 4400, and 4200 cm^−1^. These results suggested that the very slight spectral pattern differences among the second derivative spectra could indicate the influences of the casein content variations.

**Figure 6:**
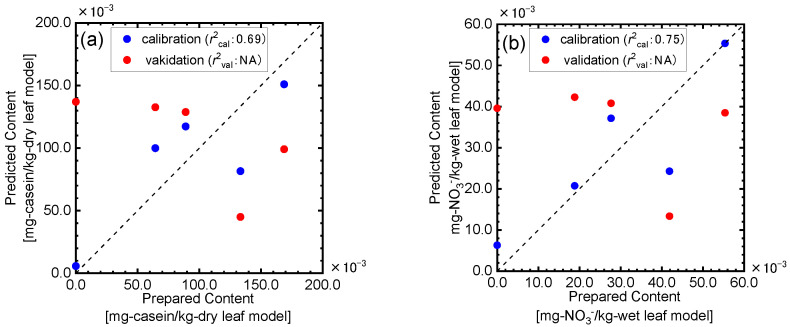
PLSR results for casein contents in (**a**) dry and (**b**) wet leaf models using spectral information in the range of 7500–4000 cm^−1^.

We then performed the PLSR analysis to determine the casein contents in the dry leaf model, in which the casein content per volume is higher than that in the wet leaf model, using the spectral information in the range of 7500–4000 cm^−1^ shown in Figure 5, and the results are displayed in Figure 6a. The best predictive model was selected as the model with the lowest mean value of absolute error (MAEtest) between the predicted and measured picking time in the LOO cross validation. The prediction of the casein contents in the dry leaf model were difficult by the PRSR method using the whole spectral in formation from 7500–4000 cm^−1^. For the wet leaf model, the casein content determination was also missed by the same method (Figure 6b). We also tried to predict the nitric acid content in the wet leaf mode using the same method, but results similar to that for the proteinic content determination were obtained. It was then too difficult to predict the nitrogen contents in the leaf models with the complicated chemical and geometrical structures by performing the PRSR analysis using the whole spectral information from 7500–4000 cm^−1^, though the high correlation coefficients between the nitrogen contents and the second derivative values were confirmed for many wavenumbers. These results experimentally suggested that the NIR spectral information could be seriously influenced by many factors such as the components other than the nitrogen ones though the nitrogen content variations could reflect the NIR spectral features and that it was important to using only the spectral information related to the nitrogen components for the prediction.

### 3.2. Extraction of NIR Spectral Bands for Quantification of Nitrogen Components

Based on the results and discussion described in Section 3.1, we then tried to extract the NIR spectral information directly characterizing the nitrogen components in the leaf models with the high correlation coefficients between the nitrogen contents and the second derivative values to determine the contents, since the NIR spectral information could contain the secondary chemical and structural information other than the target components. We then performed a two-dimensional spectroscopic analysis between the NIR spectroscopic information of the leaf models and the MIR spectral information of the nitrogen aqueous solutions since the fundamental vibrational modes are observed in the MIR region and the aqueous solution is geometrically and chemically homogeneous.

**Figure 7:**
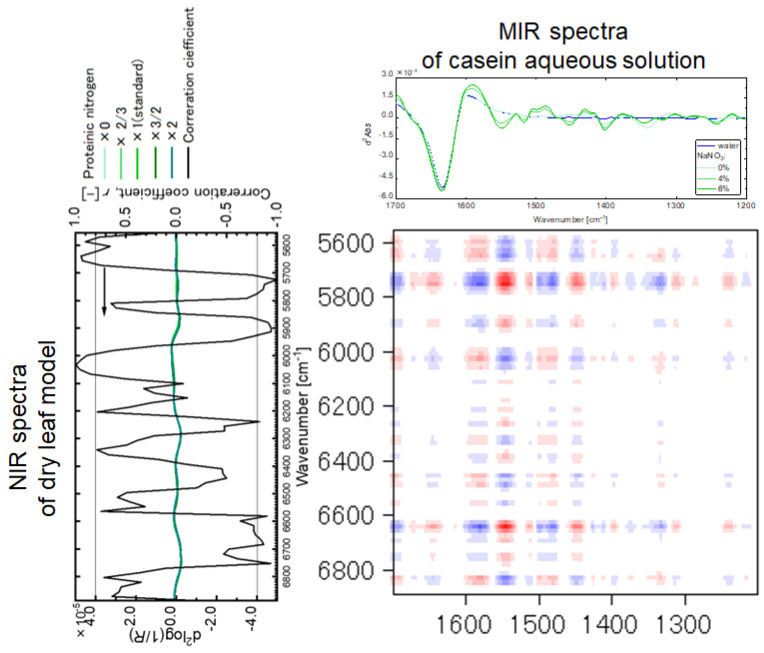
Two-dimensional spectroscopic correlation analysis between the NIR spectra of the dry leaf model and the MIR spectra of casein in the aqueous solution. The red and blue parts respectively indicate that the NIR spectra are correlated in the same directions as the MIR spectra and that the second derivatives of the MIR spectra are increasing in the negative directions.

By dividing the NIR region of 7500–4000 cm^−1^ into five regions of 7500–6890 cm^−1^, 6890–5553 cm^−1^, 5553–5142 cm^−1^, 5142–4439 cm^−1^, and 4439–4000 cm^−1^, the two-dimensional spectroscopic correlation was separately performed; Figure 7 shows the results of the two-dimensional spectroscopic correlation analysis between the NIR spectra of the dry leaf model in the region of 6890–5553 cm^−1^ and the MIR spectra of casein in the aqueous solution. The red parts indicate that the NIR spectra are correlated in the same directions as the MIR spectra. On the other hand, the blue parts indicate that the second derivatives of the MIR spectra are increasing in the negative directions, while the those of the NIR spectra are increasing in the positive directions. Also, the color depth indicates the strength of the correlation. Many correlations were confirmed for the stable absorption bands of casein in the MIR region.

**Figure 8:**
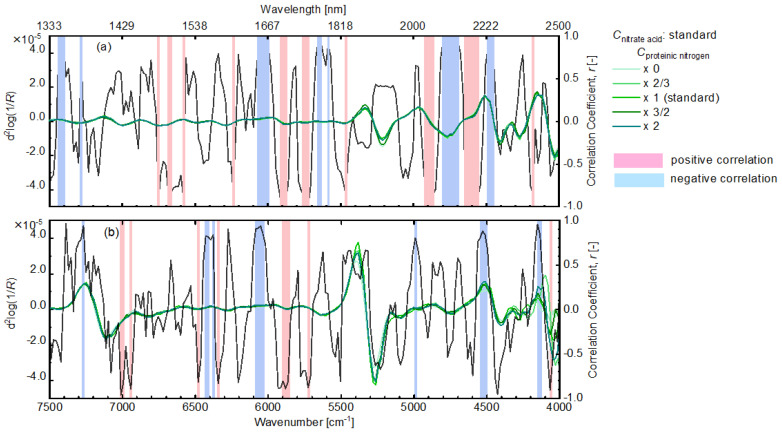
Selected bands with an absolute value of 0.8 or more for the correlation coefficients between the second derivative values of the NIR absorption spectra of the dry (**a**) or wet leaf models (**b**) and the casein contents.

**Figure 9:**
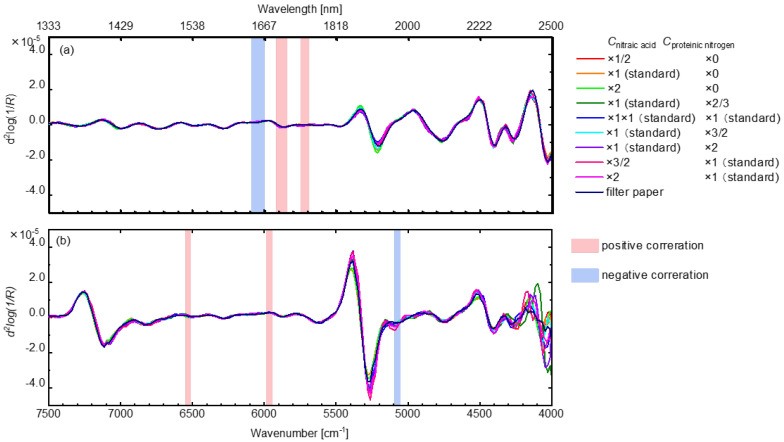
Selected bands with an absolute value of 0.8 or more for the correlation coefficients between the second derivative values of the NIR absorption spectra of the dry (**a**) or wet leaf models (**b**) and the nitric acid contents.

Additionally, among the absorption bands of casein obtained by the two-dimensional spectral correlation analysis (Figure 7), the bands with the absolute value of 0.8 or more for the correlation coefficients between the second derivative values of the NIR absorption spectra of the dry leaf models and the casein contents (Figure 5) were selected (Figure 8a). These absorption bands could characterize casein and have high correlations in the dry leaf model with chemically and geometrically complicated structures. For the wet leaf model, the same data processing was carried out; the selected bands are displayed in Figure 8b. The selected band differences between Figure 8a,b could depend on the influences of water in the wet leaf model on the NIR spectral features. Furthermore, the common bands were selected for the dry and wet leaf models (Figure 8a) to extract the casein information from the leaf models with various water contents. The same treatment was performed for nitric acid, and the absorption band was obtained (Figure 9a). It was postulated that the NIR spectroscopic band shown in Figure 8 and Figure 9 could molecularly relate to the proteinic and nitrate nitrogen and the influences of the water content and the complexities of the paper filter on the NIR spectroscopic feature are negligible. Moreover, such types of absorption bands of the proteinic and nitrate nitrogen could be useful for NIR spectroscopic quantification.

### 3.3. NIR Spectroscopic Determination of Proteinic and Nitrate Nitrogen Contents

#### 3.3.1. Prediction of Proteinic and Nitrate Nitrogen Contents in the Dry Leaf Model

**Figure 10:**
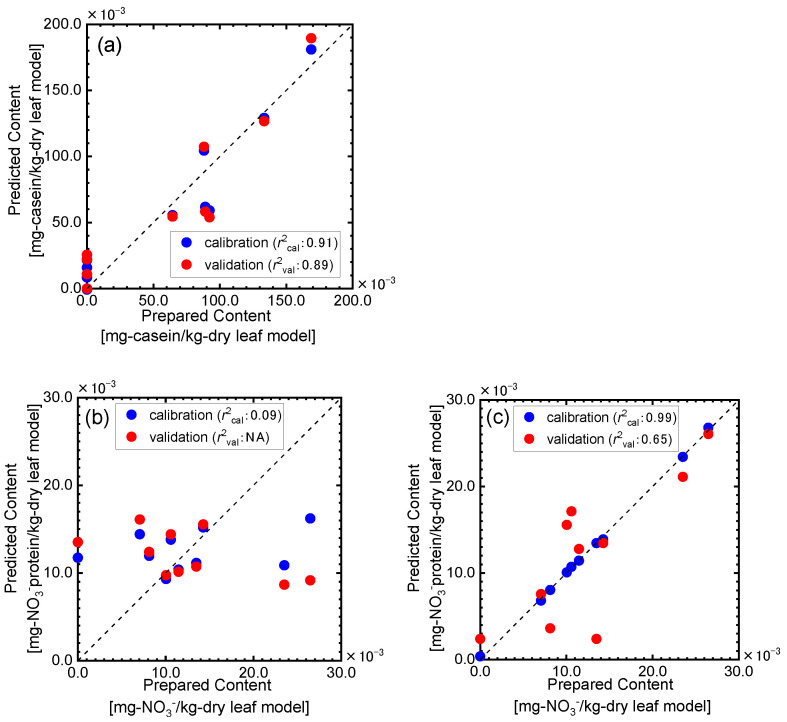
PLSR analysis results for the prediction of casein (**a**) and nitric acid (**b**,**c**) contents in the dry leaf model.

The PLSR analysis was performed using the NIR spectral information for the absorption bands selected in the above discussion to determine the proteinic nitrogen contents. As shown in Figure 10a, the casein contents in the dry leaf model, in which the casein content per volume is higher than that in the wet leaf model, could be successfully predicted with the validation determination coefficient of 0.89. On the other hand, almost no correlation was observed between the nitrate acid content in the dry leaf model predicted by the PLSR analysis using the spectral information in the selected bands (Figure 9b) and the prepared ones (Figure 10b). The selected bands indicated in Figure 9b are all very narrow, and this might mean too few data points for the PLSR analysis. We then performed the PLSR analysis using the spectral information obtained by extending the bandwidth shown in Figure 9b before and after by one point; the results are displayed in Figure 10c. By extending the bandwidth and using more spectral data points, the prediction results became better and good agreements were observed between the prepared and predicted nitric acid concentrations in the dry leaf models. These results suggested that the selected bands could be fundamentally effective for the determination of the nitrate acid content.

#### 3.3.2. Prediction of Proteinic and Nitrate Nitrogen Contents in the Wet Leaf Model

**Figure 11:**
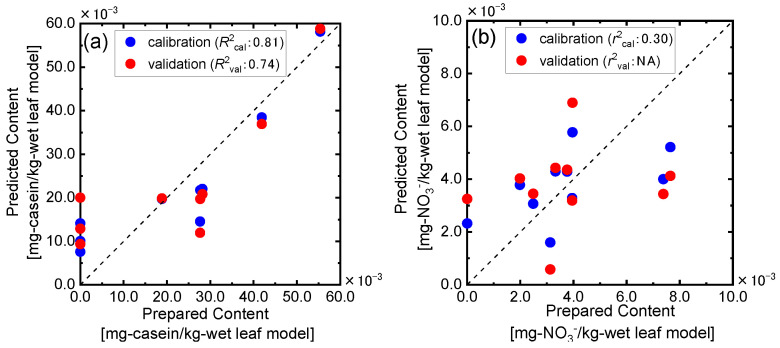
PLSR analysis results for prediction of casein (**a**) and nitric acid (**b**) contents in the wet leaf model.

For the wet leaf models in which the nitrogen components in the dry leaf models are diluted and the influence of water on the spectral features could be considered, the PLSR analysis was performed using the NIR spectral information by the method described above. As shown in Figure 11a, the predicted casein contents in the wet leaf models well agreed with the actual value using the validation determination coefficient of 0.74, which was lower than that for the dry leaf model but could be acceptable for the application as a rapid, easy, and non-chemical measurement in the agricultural fields. On the other hand, almost no correlation was observed between the nitrate acid content in the wet leaf model predicted by the PLSR analysis using the spectral information for the same bands used for the dry leaf models (Figure 11b), though good prediction results were obtained for the dry leaf models (Figure 10b). For both the proteinic and nitric nitrogen content determinations, the prediction accuracies decreased by adding water to the leaf models. These results meant that the nitrogen contents were diluted by adding water to the dry leaf model and that the spectral features could become more complex. In particular, for the nitric acid content determination, the influences were very significant. However, since the nitric acid contents in the dry leaf models were well predicted by the provided NIR spectroscopic analytical method as shown in Figure 10b, the nitrate nitrogen equivalent to the concentration in fresh leaves could be quantified based on the NIR spectroscopic information. These results suggested that a further analysis using the leaf models with the wide concentration ranges of the proteinic and nitric concentrations was necessary for constructing an accurate model, though this study could provide the new NIR spectroscopic determination method based on the fundamental vibrational mode information of the target components in chemically and structurally complicated samples such as plants.

## 4. Conclusions

By focusing our attention on the nitrogen components in plants, which are important for cultivation management in data-driven agriculture, we developed a simple, rapid, non-chemical and simultaneous quantification method for nitrate and protein nitrogen in leaf models based on NIR spectroscopic information obtained using the compact FT-NIR spectrometer system. Based on the NIR spectra of wet leaf models impregnated with protein–nitric acid mixed solutions, the dried leaf models, and the information provided by the two-dimensional analysis between the NIR spectroscopic information of the leaf models and the MIR spectroscopic information of the proteinic and nitrate nitrogen mixture solutions, the NIR bands for proteinic and nitrate nitrogen determination were extracted from the total NIR spectral information. By performing the PLSR using the selected absorption bands, the multiple modes of nitrogen in the leaf models with the chemically and geometrically complicated structure could be predicted by the diffuse NIR spectral method. In addition, it was necessary to further investigate the influences of the water content on the NIR spectral information and of the band width used for quantification. Consequently, this study plays a very important role for the application of the compact MEMS FT-NIR to obtain the bioinformation of crops on-site and for the NIR spectral image analysis of plants.

## Data Availability

The raw data supporting the conclusions of this article will be made available by the authors on request.
